# GLP-1 Agonists’ Effect on Infection and Union after Tibiotalar Fusion, Subtalar Fusion, Triple Arthrodesis

**DOI:** 10.1177/10711007251328370

**Published:** 2025-03-31

**Authors:** Michael F. Levidy, Sohrab Vatsia, Scott Tucker, Nicholas Rowe, Ashlee Macdonald, Michael Aynardi

**Affiliations:** 1 Department of Orthopaedics & Rehabilitation, Penn State Milton S. Hershey Medical Center, Hershey, PA, USA

**Keywords:** fusion, diabetes, GLP-1 agonists

## Abstract

**Background:**

GLP-1 agonist use has increased due to the rising prevalence of obesity and diabetes mellitus. Foot and ankle surgeons provide orthopaedic care to diabetic patients. The effects of GLP-1 agonists on foot fusion outcomes are not well explored.

**Methods:**

The TriNetX Global Collaborative Network was queried using *CPT* codes for patients with diabetes mellitus undergoing open tibiotalar fusions, subtalar fusions, and triple arthrodesis. Outcomes were assessed at 1 year after index surgical procedure. Cohort balancing was performed according to age at procedure, race, sex, and nicotine dependence, for body mass index, glycated hemoglobin (HbA_1c_), and estimated glomerular filtration rate (eGFR). Statistical significance was set at *P* <.05 with associated 95% CIs.

**Results:**

Two cohorts of 783 patients undergoing tibiotalar, subtalar, or triple arthrodesis fusions were compared using *CPT* coding in this study (Table 1). Among patients treated with GLP-1 agonists, the overall rate of postoperative pseudarthrosis was found to be lower (15.9% vs 20.2%, *P* = .0129, RR 1.26 (1.02-1.56). Comparison of constituent procedures in isolation showed a lower rate of pseudarthrosis for patients using GLP-1 agonist than matched controls: subtalar fusion (17.2% vs 23.4%, *P* = .0292, RR 1.36, 95% CI 1.03-1.79) and triple arthrodesis (12.4% vs 21.9%, *P* = .0120, RR 1.76, 95% CI 1.12-2.76). No significant difference was found in rates of pseudarthrosis after tibiotalar fusion (19.8% vs 21.5%, *P* = .5692, RR 1.09, 95% CI 0.81-1.46). No difference was detected in postoperative infection rates for any of the procedure types (Table 3).

**Conclusion:**

Results suggest a previously unreported better fusion rates associated with GLP-1 agonist use after fusion procedures of the foot and ankle. This study also shows no clear risk or benefit associated with GLP-1 agonists with respect to postoperative infection. Additional clinical studies are needed to clarify this association.

**Level of Evidence:** Level III, database cohort study.

## Introduction

Glucagon-like peptide 1 (GLP-1) receptor agonists are commonly used to treat diabetes and obesity and to decrease the risk of heart attack and stroke.^14^ These medications work through “augmentation of hyperglycemia-induced insulin secretion and suppression of glucagon secretion at hyper- or euglycemia.”^10^ Drugs within the class have the same underlying mechanism of action, however, various GLP-1 receptor agonists have varying formulations and methods of administration.^4^ Use of drugs in this category has skyrocketed, with prescriptions of semaglutide in the United States climbing to more than 8 000 000 at the end of 2022, with a greater than 300% increase in prescription volume demonstrated between Q1 2020 and Q4 2022.^16^

Despite the success and increasing popularity of these medications, there are important side effects that can affect patients perioperatively. The American Society of Anesthesiologists has issued consensus-based guidelines, which include pausing GLP-1 receptor agonists before surgery out of concern for regurgitation and aspiration in the setting of delayed gastric emptying.^8^ These medications may also play a role in bone quality: clinical trial data have demonstrated that patients taking semaglutide have a 5 times increased risk of hip and pelvic fractures in female patients and 4 times increased risk in patients older than 75 years of age (1.0% [24/2448] vs 0.2% [5/2424], 2.4% [17/703] vs 0.6% [4/663], respectively).^5^

Tibiotalar joint degeneration can result in significant pain and functional limitations. First described by Albert in 1879, ankle arthrodesis has emerged as a reliable way to achieve good clinical outcomes with widely reported rates of successful union and infection.^18^ Similarly, subtalar arthritis can be responsible for significant pain and functional limitation, with subtalar arthrodesis providing reliable improvements in pain and function. It is understood that poor glucose control increases risk of infection and nonunion in the setting of ankle and/or hindfoot arthrodesis. Therefore, in the setting of increasing use of GLP-1 receptor agonists, and the lack of literature examining the potential impact on orthopaedic procedures, we examine rates of infection and nonunion in patients treated with GLP-1 agonist therapy.

## Methods

The TriNetX Databse was chosen for its wide inclusion of more than 100 Healthcare Organizations (HCOs) encompassing main and satellite locations, academic and nonacademic settings, and pharmacy data. Data are prospectively collected and deidentified by the HCO via their Electronic Medical Record and shared with TriNetX. TriNetX was queried for patients with an underlying diagnosis of type 2 diabetes mellitus who underwent tibiotalar fusion, subtalar fusion, and triple arthrodesis from 2005 to 2024. Arthroscopic procedure codes were excluded to limit experimental variables and reduce confounding. Pantalar fusions were excluded due to a much smaller sample size (n < 100) relative to the included procedures. *Current Procedural Terminology* (*CPT*) coding was used to query procedure types (Table 1). *International Classification of Diseases* (*ICD*) coding was not used because it lacks unique codes to distinguish Subtalar Fusion and Triple Arthrodesis in the TriNetX system. Patients were split into cohorts based on whether they had been prescribed or represcribed GLP1-agonist medications up to 1 year before surgery. Medication data was queried using TriNetX medication data codes for the following medications: tirzepatide, semaglutide, lixisenatide, dulaglutide, liraglutide, and pramlintide.

**Table 1.** *CPT* Codes Queried and Their Corresponding Procedures.

Procedure	*CPT*
Tibiotalar fusions	27870
Subtalar fusion	28725
Triple arthrodesis	28715

Cohorts were propensity-matched 1:1 according to a greedy-nearest-neighbor matching algorithm with a caliper of 0.1 pooled SDs. Categorical variables included in the model were age at procedure, race, sex, and nicotine dependence. Body mass index (BMI), glycated hemoglobin (HbA_1c_), and estimated glomerular filtration rate were used to balance the cohort for severity of diabetes. Cohorts were balanced for these variables based on the categorizations in Table 2. Outcomes of interest in this study were evaluated at 1 year and included failure to achieve fusion and postsurgical infection. Failure to achieve fusion was defined as those patients receiving a new diagnosis of *ICD-10* code M96.0, pseudarthrosis after fusion, or arthrodesis. Postsurgical infection was defined as those patients with a new diagnosis of *ICD-10* code T81.4, infection following a procedure.

**Table 2.** Groupings of Continuous Variables for Cohort Matching.

BMI	HbA_1c_	eGFR
0-20	0-5.6	0-15
21-25	5.6-6.5	15-30
26-30	6.6-7.5	30-45
31-35	7.6-8.5	45-60
36-40	8.6-9.5	60-90
40+	9.5+	90+

Abbreviations: BMI, body mass index; eGFR, estimated glomerular filtration rate; HbA_1c_, glycated hemoglobin.

The percentages of patients from each cohort who developed these outcomes were compared for each procedure type. Subsequently, a combined analysis was performed analyzing outcomes after any of the 3 procedures (tibiotalar fusion, subtalar fusion, and triple arthrodesis). The TriNetX platform software was used to run χ^2^ tests for statistical significance. Statistical significance was set at *P* <.05 with associated 95% CIs.

## Results

Two cohorts of 783 patients undergoing tibiotalar, subtalar, or triple arthrodeses fusions were compared using *CPT* coding in this study (Table 1). Among patients treated with GLP-1 agonists, the overall rate of postoperative pseudarthrosis was found to be lower (15.9% vs 20.2%, *P* = .0129; relative risk [RR] 1.26, 95% CI 1.02-1.56). Comparison of constituent procedures in isolation showed a lower rate of pseudarthrosis GLP-1–receiving patients who underwent subtalar fusion (17.2% vs 23.4%, *P* = .0292; RR 1.36, 95% CI 1.03-1.79) and triple arthrodesis (12.4% vs 21.9%, *P* = .0120; RR 1.76, 95% CI 1.12-2.76). No significant difference was found in rates of pseudarthrosis after tibiotalar fusion (19.8% vs 21.5%, *P* = .5692; RR 1.09, 95% CI 0.81-1.46). No difference was detected in postoperative infection rates for any of the procedure types (Table 3). Additional data on patient demographic breakdown is provided in Table 4.

**Table 3.** Outcomes After Fusion Procedures Queried Using *CPT* Codes.

*CPT* Only	Cohort Size, n	Pseudarthrosis, n (%)	Infection, n (%)
Tibiotalar			
Without GLP-1	339	73 (21.5)	43 (12.7)
With GLP-1	339	67 (19.8)	42 (12.4)
* P*		.5692	.9077
RR (95% CI)		1.09 (0.81, 1.46)	1.02 (0.65, 1.62)
Subtalar			
Without GLP-1	406	95 (23.4)	42 (10.3)
With GLP-1	406	70 (17.2)	50 (12.3)
* P*		**.0292**	.3758
RR (95% CI)		1.36 (1.03, 1.79)	0.84 (0.57, 1.24)
Triple			
Without GLP-1	201	44 (21.9)	18 (8.9)
With GLP-1	201	25 (12.4)	16 (7.9)
* P*		**.0120**	.72
RR (95% CI)		1.76 (1.12, 2.76)	1.13 (0.59, 2.14)
Tibiotalar or subtalar or triple			
Without GLP-1	783	158 (20.2)	73 (9.3)
With GLP-1	783	125 (15.9)	77 (9.8)
* P*		**.0129**	.73
RR (95% CI)		1.26 (1.02, 1.56)	0.83 (0.67, 1.32)

Abbreviations: *CPT*, *Current Procedural Terminology*; GLP-1, glucagon-like peptide 1; RR, relative risk.

Boldface indicates significance (*P* < .05).

**Table 4.** Patient Demographics.

	Without GLP-1				With GLP 1				
Characteristic	Patient Count	Percent	Mean	SD	Patient Count	Percent	Mean	SD	*P* Value
Age at index	783	100	57.15	12.05	783	100	57.74	9.93	.29
White	568	72.54			566	72.29			.91
Male	379	48.40			371	47.38			.69
Black or African American	126	16.09			119	15.20			.63
Hispanic or Latino	78	9.96			76	9.71			.87
Asian	10	1.28			10	1.28			>.99
**Medical comorbidities**									
Chronic ischemic heart disease	164	20.95			173	22.10			.58
Personal history of nicotine dependence	151	19.29			149	19.03			.90
Peripheral vascular disease	116	14.82			118	15.07			.89
Chronic obstructive pulmonary disease	58	7.41			71	9.07			.23
Fibrosis and cirrhosis of liver	25	3.19			23	2.94			.77
eGFR	605	77.27	69.16	30.20	591	75.48	70.12	29.32	.58
0-30	144	18.39			139	17.75			.74
30-60	302	38.57			310	39.59			.68
60-90	403	51.47			425	54.28			.27
At least 90	258	32.95			271	34.61			.49
BMI	591	75.48	37.26	8.01	612	78.16	37.52	7.52	.56
0-20	17	2.17			16	2.04			.86
20-30	152	19.41			164	20.95			.45
30-40	370	47.25			397	50.70			.17
≥40	294	37.55			305	38.95			.57
HbA_1c_	548	69.99	7.44	1.75	540	68.97	7.31	1.73	.24
0%-5.6%	45	5.75			61	7.79			.11
5.6%-6.5%	221	28.23			241	30.78			.27
6.5%-7.5%	291	37.17			301	38.44			.60
7.5%-8.5%	262	33.46			252	32.18			.59
≥8.5%	274	34.99			277	35.38			.87

Abbreviations: BMI, body mass index; eGFR, estimated glomerular filtration rate; GLP-1, glucagon-like peptide 1; HbA_1c_, glycated hemoglobin.

## Discussion

We used a retrospective database review to evaluate the impact of GLP-1 agonists on rates of infection and nonunion after tibiotalar fusion, subtalar fusion, and triple arthrodesis. Our data showed an increase in rates of union associated with those receiving GLP-1 agonists. Our data demonstrated similar rates of infection and fusion as Cardoso and Veljkovic’s.^2^ Similarly, Wong et al^17^ reported nonunion and infection rates of 16% and 6%, respectively. A 2021 systematic literature review by Oshba et al^12^ reported similar fusion rates. Overall, our patient demographics indicate a representative sample of orthopaedic patients and their comorbidities, suggesting good generalizability to many practice environments (Table 4).

Orthopaedic surgeons, particularly those operating about the foot and ankle, are well aware of the impact diabetes and glycemic control can have on postsurgical outcomes. Previous studies have demonstrated increased complication rate and infection rate after hindfoot fusion in patients with poorly controlled diabetes.^9^ Similarly, previous work has demonstrated higher rates of complications with ankle fracture management in uncontrolled diabetics as compared to controlled diabetics.^7^ GLP-1 agonists have previously been shown to reduce perioperative glucose and insulin administration without the risk for hypoglycemia.^6^ Perhaps less well known are the immunologic and anti-inflammatory effects of GLP-1 agonists,^1^ which may raise concern for infectious risk. Concurrently, there is a growing body of evidence to suggest that GLP-1 agonists impact bone metabolism. Nuche-Berenguer et al demonstrated increases in bone-anabolic agents such as osteoprotegerin and RANKL, in addition to decreased trabecular anisotropy.^11^ Prior work demonstrates that GLP-1 agonist therapy promotes bone formation, downregulates bone resorption, and alters the balance between resorption and formation during remodeling.^19^ On a cellular level, an increase in osteoblasts and osteoclasts, but also decreased osteoclast activity, has been shown in mice receiving chronic GLP-1 therapy.^13^ These results were re-created more recently by Cheng et al,^3^ who found GLP-1 agonist therapy attenuated osteoporosis by preventing deterioration of trabecular microarchitecture and enhancing bone strength in a diabetic mouse model.

A causative relationship cannot be established based on the outcomes of this study; however, our findings may have a mechanistic underpinning. The aforementioned decrease in osteoclastic activity^19^ may leave additional bone at the fusion site. This, combined with enhanced glucose control^6^ and upregulated osteoblasts may favor bony fusion, and account for the increased rate of fusion documented in our study. This study found no difference in rates of infection despite the known benefit of GLP-1 agonists on perioperative glucose control. Countervailing phenomena associated with GLP-1 agonists may play a role here - improved glucose control may reduce infectious risk, but other effects of GLP-1 agonists, namely, a previously documented antiinflammatory and possibly immunomodulatory effect,^1^ may offset it. Additional research is needed to parse the exact effect these medications make on the immune system and operative infectious risk independent of their effects on glucose control.

This is a retrospective database study and therefore limited by the accuracy of inputted coding data. Notably, tibiotalar fusions did not significantly differ between GLP-1–treated and GLP-1-naïve patients. This may be a result of cross-coding arthroscopic vs open procedures leading to a higher variance in outcomes. The relative popularity of the arthroscopic approach to tibiotalar fusion may have made this procedure particularly vulnerable to confounding. Another limitation of this study is the inability to assess individual patients imaging or functional status for indicators of fusion quality, such as subsequent refracture through the fusion site. Further limitation is introduced by the spectrum of diagnostic parameters that spur providers to enter a database-discoverable pseudarthrosis code—some may rely on purely clinical factors to assess fusion, while others may prefer evaluation via radiography or CT scan. These limitations are of particular concern for patients taking GLP-1 agonists given the possible attenuation of bone remodeling at the fusion cite via downregulated osteoclast activity. Another limitation is conflicting indications for GLP-1 usage. The data collection period for this study straddles the 2015 FDA approval for a weight loss indication, before this, GLP-1 agonists were only indicated for diabetic control. Different dosages of GLp-1 agonists are used for different indications, and may confound the results. Similarly, duration of GLP-1 therapy may confound results. In this study, we included patients who had been prescribed GLP1 therapy between 1 week and 1 year of surgery. Although GLP-1 medications have been shown to impact blood glucose in days to a week,^15^ it is possible that effects on bony metabolism take longer to manifest. The role of better glucose control and weight control based on duration of exposure to GLP-1 agonists may also confound outcomes and pose limitations to our findings.

## Conclusion

In this propensity-matched database study we found that patients taking GLP-1 agonists were associated with increased rates of fusion after undergoing subtalar, and triple arthrodesis. Analysis of combined procedures similarly showed an increased rate of fusion in GLP-1 taking patients. No differences in rate of infection were found for any of the procedure types. Additional prospective trial research is needed to fully elucidate the potential impact of GLP-1 agonists on bony healing and postoperative outcomes.

## Supplemental Material

sj-pdf-1-fai-10.1177_10711007251328370 – Supplemental material for GLP-1 Agonists’ Effect on Infection and Union after Tibiotalar Fusion, Subtalar Fusion, Triple Arthrodesis Supplemental material, sj-pdf-1-fai-10.1177_10711007251328370 for GLP-1 Agonists’ Effect on Infection and Union after Tibiotalar Fusion, Subtalar Fusion, Triple Arthrodesis by Michael F. Levidy, Sohrab Vatsia, Scott Tucker, Nicholas Rowe, Ashlee Macdonald and Michael Aynardi in Foot & Ankle International
